# miR-1247-3p regulation of CCND1 affects chemoresistance in colorectal cancer

**DOI:** 10.1371/journal.pone.0309979

**Published:** 2024-12-31

**Authors:** Dequan Wang, Jielian Wang, Fei Yao, Zhufu Xie, Jianze Wu, Huiguang Chen, Qingming Wu

**Affiliations:** 1 Hubei Province Key Laboratory of Occupational Hazard Identification and Control, Institute of Infection, Immunology and Tumor Microenvironment, School of Medicine, Wuhan University of Science and Technology, Wuhan, Hubei, China; 2 Department of Internal Medicine, Tianyou Hospital, Wuhan University of Science and Technology, Wuhan, China; 3 College of Health Medicine, China Three Gorges University, Yichang, Hubei, P.R. China; 4 Department of Gastroenterology, Tianyou Hospital, Wuhan University of Science and Technology, Wuhan, Hubei, China; University of the Punjab, PAKISTAN

## Abstract

The effectiveness of chemotherapy involving 5-fluorouracil and cisplatin (DDP) for the treatment of colorectal cancer (CRC) is often limited due to the emergence of drug resistance. An increasing body of research highlights the crucial role of abnormally expressed microRNAs (miR/miRNAs) in fostering drug resistance in various types of cancer. The present study was the first to explore the potential roles and mechanisms of the small non-coding RNA miR-1247-3p in CRC, particularly its association with DDP resistance in CRC. The findings of the current study revealed a significant decrease in miR-1247-3p expression in CRC cells, especially those resistant to drugs. By contrast, there was a marked increase in the expression of cyclin D1 (CCND1), a known target gene of miR-1247-3p that is negatively regulated by this miRNA. By modulating CCND1, miR-1247-3p can effectively reduce drug resistance and promote apoptosis in CRC cells, suggesting that miR-1247-3p could potentially reduce chemotherapy resistance by targeting CCND1. These results highlight the pivotal role of miR-1247-3p in reducing chemotherapy resistance through the inhibition of CCND1, providing insight into a promising therapeutic strategy for overcoming CRC resistance.

## Introduction

Colorectal cancer (CRC) is one of the most common cancers globally, with increasing rates of morbidity and mortality, and a poor prognosis [1, 2]. For patients with advanced CRC, adjuvant chemotherapy involving fluorouracil- and platinum-based agents is the recommended standard of care according to various treatment guidelines. However, the effectiveness of conventional chemotherapy in advanced CRC is limited, with chemoresistance being a marked factor leading to poor patient outcomes [3–6]. Chemoresistance often results from a complex combination of factors, including reduced intracellular drug accumulation, increased activity of detoxification systems, enhanced DNA damage repair mechanisms and inhibition of cell death-promoting pathways [7–10]. Among these, abnormal apoptosis refers to the ability of chemotherapeutic drugs to inhibit tumor growth by inducing apoptosis in tumor cells. When the expression of pro-apoptotic and apoptosis-inhibiting genes is abnormal in cells, they can resist cell death induced by chemotherapeutic drugs by destroying apoptotic pathways, which leads to the occurrence of drug resistance in tumors. Enhancement of DNA damage repair represents the fact that chemotherapeutic drugs can directly or indirectly lead to cellular DNA damage and changes in cell-cycle distribution. When the cellular damage repair ability is enhanced, it does not signal to the apoptosis mechanism, thus enhancing the cellular resistance to chemotherapeutic drugs. At present, research is focused on developing reversal agents to counter these mechanisms. While these efforts have shown promise in reducing tumor cell chemoresistance, significant progress in this area is still needed [11]. Therefore, there is an urgent need to fully understand the molecular mechanisms involved to develop innovative strategies for CRC treatment.

Existing evidence strongly supports the notable role of various non-coding RNAs (ncRNAs), particularly small non-coding RNAs, microRNAs (miR/miRNAs), in influencing numerous cellular processes [12, 13]. miRNAs, short non-coding RNA molecules typically comprised of 20–24 nucleotides, have been identified as critical contributors to the development of various human cancers. miRNAs function through interactions with target mRNAs, negatively regulating target gene transcription by binding to the 3’-UTR region. They are also involved in the development of drug resistance in CRC. Recent studies have highlighted the role of miRNAs such as miR-21, miR-29a, miR-125b and miR-372-3p in modulating CRC drug resistance [14–17]. These miRNAs can finely regulate the expression of genes associated with key factors such as drug metabolism, DNA repair and apoptosis, which are crucial determinants of drug resistance.

Previous studies have highlighted the abnormal expression patterns and diverse functions of miR-1247-3p in various types of cancer [18, 19]. In lung adenocarcinoma (LUAD), miR-1247-3p exhibits higher expression in normal tissue compared with cancerous tissue. Overexpression of miR-1247-3p has been shown to counteract drug resistance in LUAD. Recent research has revealed that miR-1247 activates cancer-associated fibroblasts in liver cancer metastasis. Through its effect on B4GALT3, miR-1247-3p activates the β1 integrin-NF-κB signaling pathway in fibroblasts, thereby promoting the migration and invasion of breast cancer (BC) cells. However, the exact role of miR-1247-3p in CRC and its underlying mechanisms remain unclear. The current study was the first to explore the biological functions and molecular complexities of miR-1247-3p, and its impact on drug resistance in CRC with the aim to identify promising biomarkers for improving CRC therapy in the future.

## Materials and methods

### Cell culture

The human colon cancer cell lines HCT8, the 5-fluorouracil (5-Fu)-resistant cell line HCT8/5-Fu and the cisplatin (DDP)-resistant cell line HCT8/DDP were obtained from Hunan Fenghui Biotechnology Co., Ltd. HCT8 cells were cultured in RPMI-1640 medium (Gibco; Thermo Fisher Scientific, Inc.) supplemented with 10% FBS (Gibco; Thermo Fisher Scientific, Inc,) and 100 U/ml penicillin/streptomycin (Gibco; Thermo Fisher Scientific, Inc,) at 37°C with 5% CO_2_. When the cell density of HCT8 cells reached ~8x10^5^ cells/ml, they were transferred to a complete medium containing 500 ng/ml 5-Fu for further proliferation. During the passaging process, the drug concentration was gradually increased, usually by 1-2-fold increments, while carefully monitoring and maintaining optimal cell conditions and stable growth. The highest drug concentration was consistently kept at 5 μg/ml. If cell growth slowed down, a temporary switch to a 5-Fu-free complete medium was carried out until cell growth improved, at which point the necessary 5-Fu concentration was re-introduced. The culture conditions for HCT8/DDP cells were similar to those of HCT8/5-Fu cells with an initial drug concentration of 100 ng/ml and a final concentration of 1 μg/ml, following the established protocol. 293FT cells (cat. no. CL-0313; Procell Life Science & Technology Co., Ltd.) were cultured in DMEM (Gibco; Thermo Fisher Scientific, Inc,) supplemented with 10% FBS and 1% penicillin/streptomycin.

### Bioinformatics analysis

The colorectal cancer miRNA sequencing datasets GSE190951 [20] and GSE128446 [21] were searched through the GEO database, and the differential miRNAs in them were screened using GEO2R, with the thresholds for differential expression set at log2 fold change |logFC| > 1 and an adjusted p-value < 0.05. The expression levels of miR-1247-3p and CCND1 in colorectal cancer tissues versus normal tissues were compared using the UALCAN database. The impact of miR-1247-3p on overall survival in colorectal cancer patients was analyzed using the ENCORI and TCGA databases. Kaplan-Meier Plotter database was used to assess the effect of CCND1 on overall survival of colorectal cancer patients. The dataset types and sample sizes are detailed in S1 Table.

### Transcriptome sequencing

Total RNA was extracted using TRIzol reagent (Thermo Fisher Scientific, Inc.) according to the manufacturer’s protocol. The number of total RNA samples was evaluated as described in our previous study [17]. Total RNA (5 μg) from each sample was used as input material for the preparation of strand-specific complementary DNA (cDNA). Strand-specific cDNA libraries were constructed as follows: Ribosomal RNA was first removed using the Ribo-Zero Magnetic kit (Epicentre; Illumina, Inc.) and then fragmented into 300-bp-long fragments using fragmentation buffer. Next, first-strand cDNA was synthesized using random hexamer primers, and dUTPs were used instead of dTTPs in the synthesis of the second-strand cDNA. Double-stranded cDNA (ds-cDNA) was isolated from the second-strand reaction mixture using AMPure XP beads, and a single ‘A’ nucleotide was added to the 3’ end of the blunt fragment. Finally, the ends of the ds-cDNA were ligated with multiple indexing adapters. A library of 300-400-bp cDNA target fragments was screened and then subjected to 15 cycles of PCR using Phusion DNA polymerase (New England BioLabs, Inc.). After quantification with a TBS-380 fluorometer (Turner Designs), the library was sequenced on the Hiseq 2000 platform (2x150-bp-long paired-end reads). For small RNA, sequencing libraries were constructed using the Truseq^™^ Small RNA Sample Prep Kit (Illumina, Inc.) following the manufacturer’s protocol. Briefly, small RNAs were ligated to sequencing adapters, cDNAs were synthesized and amplified in 12 PCR cycles to generate libraries, and then the products were purified using 6% Novex TBE PAGE gels (Thermo Fisher Scientific, Inc.). The quality of the libraries was assessed using a 2100 Bioanalyzer (Agilent Technologies, Inc.) and single-end sequencing was performed on a Hiseq 2000 platform. There were three replicates per set, and whole transcriptome sequencing was performed by Shanghai Majorbio Bio-pharm Technology Co., Ltd. Sequencing data may include reads with adaptors or those of low quality, which can significantly interfere with downstream data analysis. Therefore, further filtering of the sequencing data is necessary. The filtering criteria primarily include the removal of sequences with adaptors at the 3’ end using Cutadapt and the exclusion of reads with an average quality score below Q20. The filtered reads were compared with the reference genome using HISAT2 2.1.0 (http://ccb.jhu.edu/software/hisat2/index.shtml). miRNA expression levels were determined using the million transcripts method. If the reference genome is appropriately chosen and there is no contamination in the related experiment, the mapping ratio of sequencing reads typically exceeds 70%. In addition, DE miRNAs (differential miRNA expression) with |log2FoldChange|>1 and P<0.05 between chemo-sensitive and -resistant cell lines were identified using DESeq (Bioconductor - DESeq).

### Reverse transcription-quantitative

*PCR (RT-qPCR)*. Total RNA was carefully extracted from CRC cells using an efficient RNA extraction kit (cat. no. RC112-01; Vazyme Biotech Co., Ltd.). The concentration and purity of the RNA were assessed using a spectrophotometer (Thermo Fisher Scientific, Inc.). RT was then performed using RNA from CRC cells using a specialized kit (cat. no. FSQ-101; Toyobo Life Science). U6 was used as the internal reference for miRNA, while GAPDH was used as the internal reference for mRNA. This allowed for the effective conversion of quantified total RNA into cDNA. The primers for both upstream and downstream sequences, as well as the RT primers, were designed by Shanghai Sangong Pharmaceutical Co., Ltd., and RT was carried out according to the manufacturer’s instructions. The total reaction system was 10 μl, including 4.5 μl cDNA, 5 μl SYBR and 0.5 μl upstream and downstream primer mixtures. To start the reaction, SYBR was pre-mixed with the upstream and downstream primers and they were combined with cDNA. CT values were determined using a RT-qPCR system (Bio-Rad Laboratories, Inc.). The relative expression levels of RNAs were quantified using the 2^-ΔΔCq^ method [17]. The related primer sequences are listed in S2 Table.

### Cell transfection

miR-1247-3p mimics, miR-1247-3p inhibitors and their respective negative control (NC) mimics were obtained from Jimma Co. Ltd (Suzhou, China, https://www.genepharma.com/). These agents were transfected into HCT8, HCT8/5-Fu and HCT8/DDP cells using the Lipofectamine^™^ 2000 transfection reagent (cat. no. 11668030; Invitrogen; Thermo Fisher Scientific, Inc.). The transfection system was prepared when the cell density of the 6-well plate was 70%, and the transfection system for each well was 200 μl Opti-MEM (Gibco; Thermo Fisher Scientific, Inc,), 100 μM plasmid, and 10 μl lipo2000, centrifuged at 3500 rpm for 15 s and then left to stand for 5 min before being added to the wells drop-by-drop and incubated at 37°C with 5% CO_2_ for 48h. When the density of 293FT cells in the 6-well plate was 70%, the transfection system was prepared, and the transfection system in each well was 200 μl OPti-mem, 0.5 μg pMD2.G (cat. no. 12259; Addgene), 0.75 μg psPAX2 (cat. no. 12260; Addgene), 1 μg of the target plasmid, vortexed and mixed, centrifuged at 3000 rpm for 15s, added 6.75 μl PEI MAX (cat. no. 24765–1; Polysciences), mixed upside down, centrifuged at 3000 rpm for 15s, and left to stand for 15min, then add it drop by drop into the cell culture medium of the wells, replace it with normal medium after 12h, incubate at 37°C with 5% CO_2_ for 48h, and then collect the viral liquid with a 0.45μm filter. When the density of HCT8/5-Fu or HCT8/DDP cells was 30%-50%, the infection system was prepared, and each well of the infection system was 1 ml of complete medium, 1 ml of viral liquid, and 2 μl of Polybrene (8 μg/ml; cat. no. BL628A; Biosharp), and the configured infection system replace the old medium, incubated at 37°C with 5% CO_2_ for 48 h. Stable expression cell lines were obtained after 48 h of incubation with complete medium containing 6 μg/ml (HCT8/5-Fu) and 16 μg/ml (HCT8/ DDP) puro (cat. no. BS111; Biosharp). The related plasmid sequences are listed in S3 and S4 Tables.

### MTT assay

HCT8/5-Fu cells, HCT8/DDP cells or HCT8 cells were transfected with 100 nM of either NC mimic, miR-1247-3p mimic, or inhibitor. After incubating for 48 h, these transfected cells were seeded into 96-well plates at a density of 5x10^3^ cells/well. After incubating for another 24 h in a controlled incubator, the appropriate drug concentration was added. Following additional incubation, 100 ml MTT (0.5 mg/ml) solution was added to every well, and the plates were incubated for 4 h in the incubator. Subsequently, 100 μl of dimethyl sulfoxide was added, and absorbance was measured at 490 nm using a multifunctional enzyme marker.

### Flow cytometry

Following transfection of HCT8/5-Fu cells, HCT8/DDP cells, or HCT8 cells with miR-1247-3p mimics and inhibitors, the cells were subjected to treatment with 50 μg/ml 5-Fu or 5 μg/ml DDP for 48 H, enabling the observation of the impact of miR-1247-3p on apoptosis and drug sensitivity. Apoptosis was assessed through Annexin V/PI staining, followed by flow cytometry. The acquired data were subjected to comprehensive analysis using FlowJo VX. To evaluate cell cycle progression, the transfected cells were harvested and fixated using pre-chilled 70% anhydrous ethanol overnight. After two washes with PBS, a mixture consisting of 196 μl PBS, 2 μl Triton X-100 and 2 μl RNAase was introduced. Following a 30-min incubation in a 37°C water bath, 5 μl PI staining solution was added, and the samples were incubated for an additional 30 min in the dark. Flow cytometry was then used to ascertain alterations in the cell cycle.

### Western blotting

CRC cells were subjected to RIPA lysis buffer (cat. no. P0013B; Yeasen Biotechnology (Shanghai) Co., Ltd.) to extract cellular protein. The resulting lysates were disrupted through ultrasonication and subsequently centrifuged at 16020 xg for 20 min at 4°C. Protein concentrations were determined using the BCA kit (cat. no. 20201ES86; Yeasen Biotechnology (Shanghai) Co., Ltd.), with a fixed sample volume of 20 μg and a sample volume of 10 μl. Samples were electrophoresed in an acrylamide gel at 80 V for 40 min, followed by electrophoresis at 100 V for 60 min. And then wet rotated at 100 V for 100 min in an ice water bath. The polyvinylidene difluoride (PVDF) membrane was washed with TBST (0.1%Tween-20) for 10 min, and this was repeated three times after being incubated in 5% skimmed milk for 1 h. The membrane was incubated with primary antibody overnight at 4°C. After thorough washing with TBST, the membrane was further incubated with a horseradish peroxidase-labeled antibody (1:5,000; cat. no. AS014; ABclonal Biotech Co., Ltd.) for 1 h at room temperature. Subsequently, the protein bands were visualized and detected using the ECL Luminescent Substrate Detection Kit (cat. no. PMK0448; Bioprimacy, http://www.biopmk.com/) following a 1-min incubation. Cleaved-caspase3 monoclonal antibody was purchased from Cell Signaling Technology, Inc. (cat. no. 9661). Cyclin D1 (CCND1) monoclonal antibody was purchased from ABclonal Biotech Co., Ltd. (cat. no. A19038). Active-caspase3 monoclonal antibody was purchased from ABclonal Biotech Co., Ltd. (cat. no. A19654).

### Dual luciferase reporter gene assay

The wild-type (WT) and mutant (MUT) plasmids of CCND1 mRNA 3’ UTR were constructed, and when the density of 293FT cells was 70%, Lipofectamine^™^ 2000 was used to transfect CCND1 WT + miR-1247-3p mimics, CCND1 WT + NC mimics, CCND1 MUT + miR-1247-3p mimics, CCND1 MUT + NC mimics, replaced with normal medium after 12 h, and detected at 24 h using a dual luciferase reporter gene detection kit, and a multifunctional zymography for fluorescence intensity. The related primer sequences are listed in S5 Table.

### Statistical analysis

Data analysis was carried out using SPSS (version 26.0; IBM Corp.). Descriptive statistics, including mean and standard deviation, were employed to characterize the measurement data. To assess differences between the means of two samples, an unpaired t-test was used. Statistical significance for more than two groups was determined using one-way analysis of variance (ANOVA) following a Tukey multiple comparison post-test. P<0.05 was considered to indicate a statistically significant difference. Data visualization and further analysis were carried out using Graphpad Prism (version 9; Dotmatics).

## Results

### Low miR-1247-3p expression was associated with poor prognosis and drug resistance in CRC

**Fig 1:**
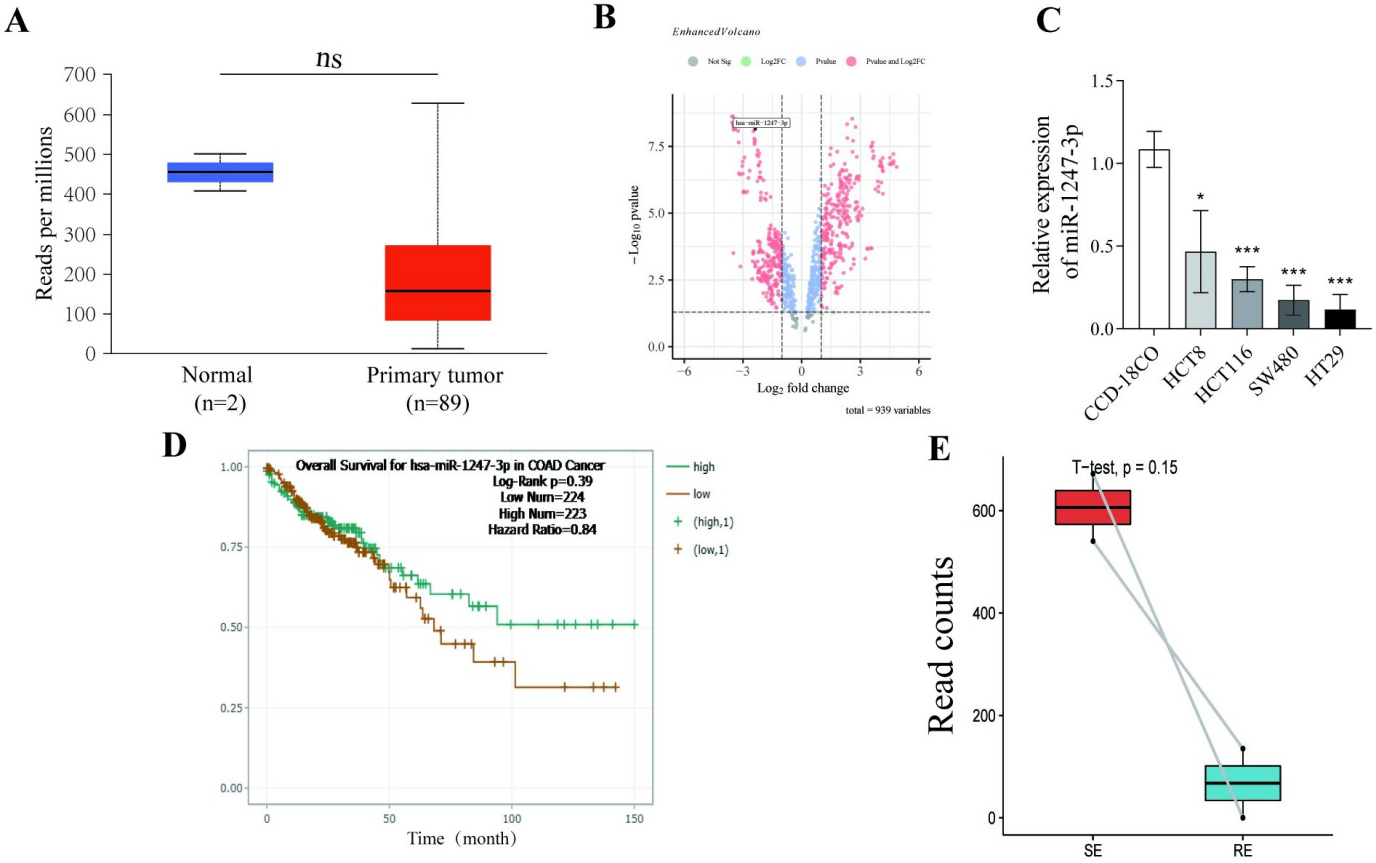
miR-1247-3p was downregulated in tissues of patients with CRC and was associated with prognosis and drug resistance. (A) and (B) miR-1247-3p expression levels in CRC tissues based on the University of ALabama at Birmingham CANcer data analysis portal and NCBI databases. (C) The effect of miR-1247-3p on overall survival of patients with CRC was analyzed based on the ENCORI database. (D) Expression of miR-1247-3p in tumor cells from patients with drug-resistant and -sensitive CRC according to the NCBI dataset GSE190951. CRC, colorectal cancer; miR, microRNA; NCBI, National Center for Biotechnology Information.

Through comprehensive analyses using the UALCAN (https://ualcan.path.uab.edu/analysis.html) database and the NCBI Non-coding RNA array dataset (GSE128446), down-regulation of miR-1247-3p expression in CRC tissues compared with normal tissues was observed (Fig 1A and 1B). The expression of miR-1247-3p in colorectal cancer cells and normal colonic fibroblasts was assessed by RT-qPCR, revealing that miR-1247-3p is significantly downregulated in colorectal cancer cells (Fig 1C). Additionally, the ENCORI analysis revealed a trend toward prolonged overall survival (OS) in patients with higher miR-1247-3p expression levels. Transcriptomic data and survival data of colorectal cancer patients were extracted using the TCGA database, and survival analysis showed that miR-1247-3p high-expression patients had significantly longer survival (Fig 1D and S1 Fig). RNA sequencing data from the NCBI database (GSE190951) were also examined which included patients with 5-Fu resistant and sensitive. After careful analysis using GEO2R (https://www.ncbi.nlm.nih.gov/geo/geo2r/), it was shown that miR-1247-3p levels were notably lower in the cancer tissues of patients with drug-resistant CRC compared with those of patients with drug-sensitive CRC (Fig 1E). In summary, these findings collectively suggest a potential negative association between miR-1247-3p and chemoresistance in CRC.

### miR-1247-3p was expressed at low levels in drug-resistant cell lines

**Fig 2:**
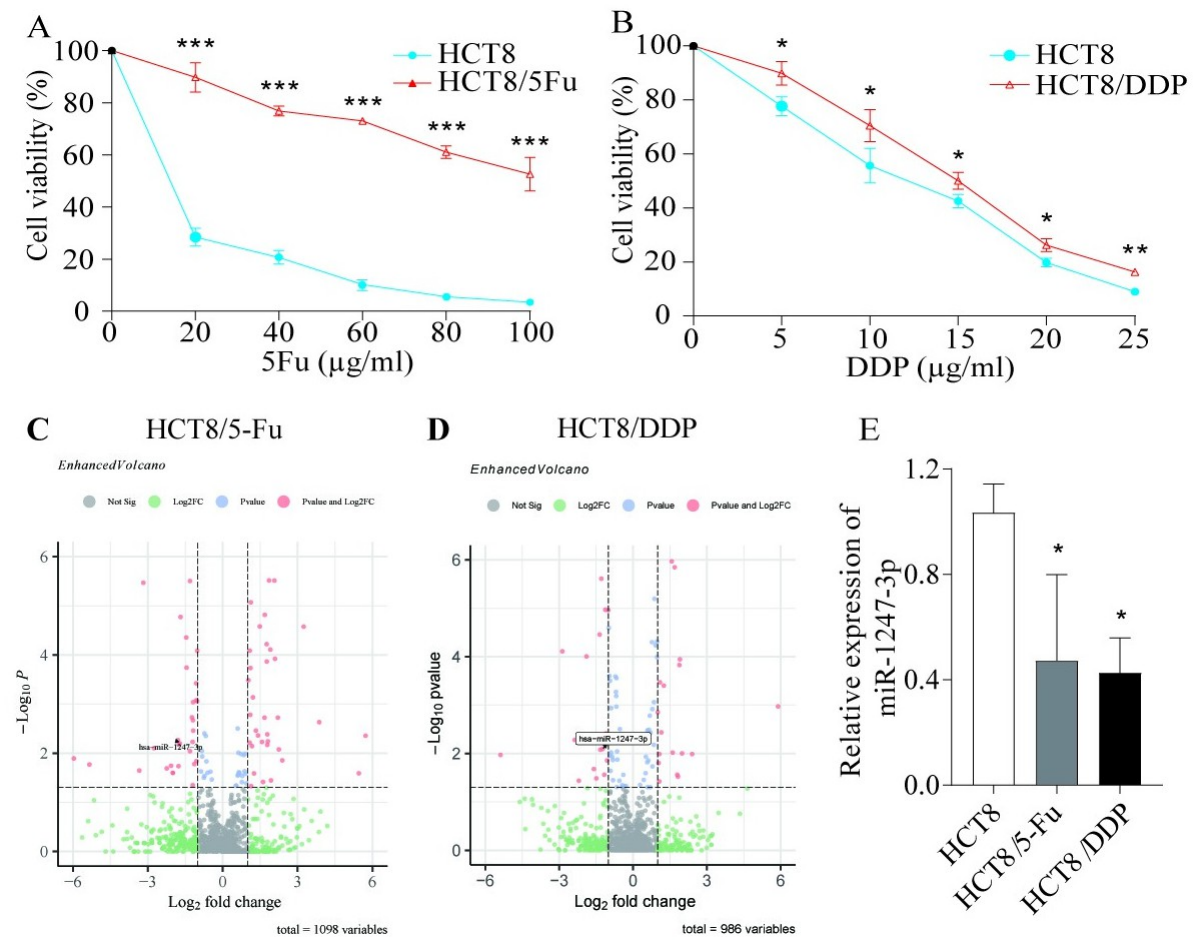
miR-1247-3p was downregulated in CRC resistant cells. (A) and (B) MTT assay to verify the drug resistance of the cells. (C) and (D) Transcriptome sequencing was performed to detect differences in miR-1247-3p expression in HCT8, HCT8/5-Fu and HCT8/DDP cells. (E) RT-qPCR was performed to verify the differential expression of miR-1247-3p in drug-resistant and parental cells. *P<0.05, **P<0.01, ***P<0.001. miR, microRNA; CRC, colorectal cancer; 5-Fu, 5-fluorouracil; DDP, cisplatin.

The drug resistance of both cell lines was validated through MTT assays (Fig 2A and 2B). The expression of miR-1247-3p was assessed in HCT8, HCT8/5-Fu and HCT8/DDP cells using both transcriptome sequencing [17] (Fig 2C and 2D and RT-qPCR (Fig 2E). The results revealed a significant reduction in miR-1247-3p levels in both HCT8/5-Fu and HCT8/DDP cells compared with the parental HCT8 cell line.

### Overexpression of miR-1247-3p promoted apoptosis and inhibited cellular chemotherapy resistance

**Fig 3:**
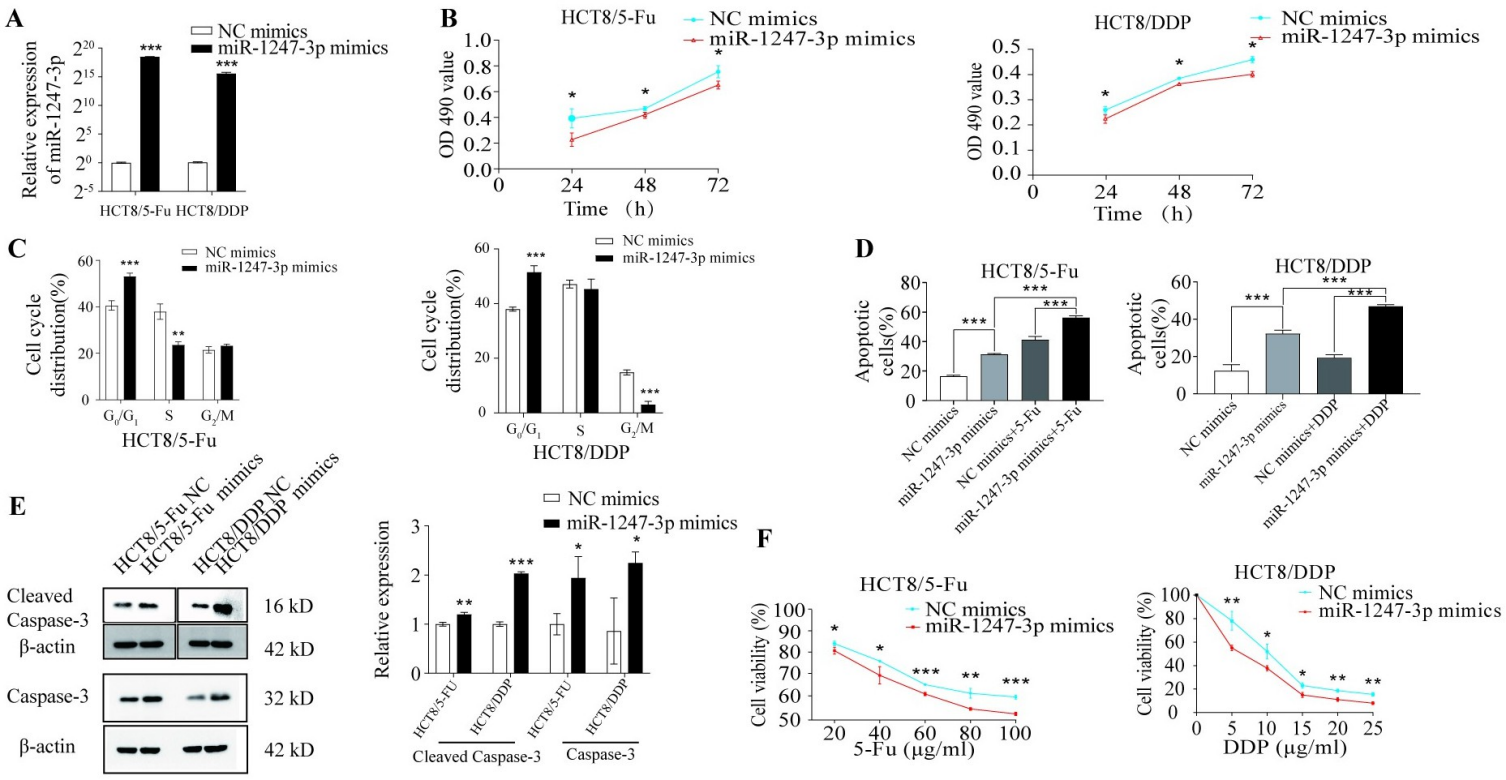
Overexpression of miR-1247-3p promoted apoptosis and inhibited resistance in drug-resistant cells. (A) HCT8/5-Fu and HCT8/DDP cells transfected with miR-1247-3p mimics or negative control. (B) An MTT assay was used to measure the proliferation of HCT8/5-Fu and HCT8/DDP cells after transfection. (C) and (D) Flow cytometry was used to analyze cell cycle and apoptosis. (E) Western blotting was used to detect the expression of apoptosis-related proteins in HCT8/5-Fu and HCT8/DDP cells after transfection. (F) An MTT assay was used to evaluate the sensitivity of HCT8/5-Fu and HCT8/DDP cells to chemotherapeutic drugs after transfection. *P<0.05, **P<0.01, ***P<0.001. miR, microRNA; CRC, colorectal cancer; 5-Fu, 5-fluorouracil; DDP, cisplatin.

Due to the observed reduction in miR-1247-3p levels in drug-resistant cells, miR-1247-3p mimics were introduced into HCT8/5-Fu and HCT8/DDP cells. The effectiveness of overexpression was confirmed via RT-qPCR (Fig 3A). With the overexpression of miR-1247-3p, a significant slowdown in cell proliferation was observed (Fig 3B), along with the induction of cell cycle arrest (Fig 3C) compared with the NC. Apoptosis increased in HCT8/5-Fu and HCT8/DDP cells transfected with miR-1247-3p mimics (Fig 3D). Western blotting further revealed an increase in the expression level of the apoptotic protein cleaved-caspase3 following miR-1247-3p overexpression (Fig 3E). Consistent with the apoptotic findings, the miR-1247-3p mimic group showed significantly increased sensitivity to chemotherapeutic drugs compared with the NC group (Fig 3F).

### Downregulation of miR-1247-3p inhibited apoptosis and suppressed cellular chemosensitivity

**Fig 4:**
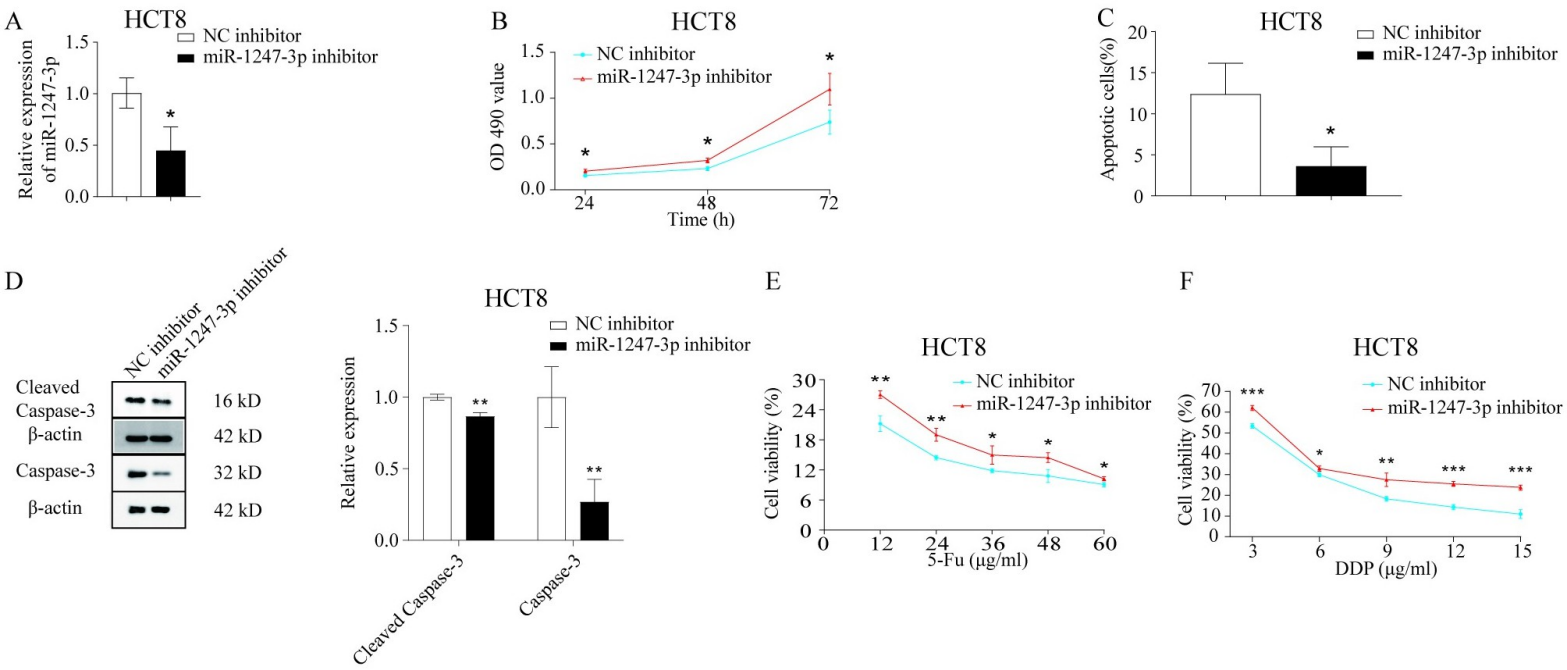
Downregulation of miR-1247-3p inhibited apoptosis and reduced chemosensitivity in cells. (A) HCT8 cells transfected with miR-1247-3p inhibitor or negative control. (B) An MTT assay was used to measure the proliferation of HCT8 cells. (C) Flow cytometry was used to analyze the apoptosis of HCT8 cells. (D) Western blotting was used to detect the expression of apoptosis-related proteins in HCT8 cells. (E) and (F) An MTT assay was used to evaluate the sensitivity of HCT8 cells to chemotherapeutic drugs. *P<0.05, **P<0.01, ***P<0.001. miR, microRNA.

To further elucidate the influence of miR-1247-3p on chemoresistance in CRC cells, miR-1247-3p was downregulated in the parental HCT8 cells. Following the downregulation of miR-1247-3p (Fig 4A), HCT8 cells exhibited increased proliferation (Fig 4B), reduced apoptosis (Fig 4C) and inhibited sensitivity to chemotherapeutic agents, as shown by western blotting (Fig 4D) and the MTT assay (Fig 4E and 4F).

### CCND1 is a target gene of miR-1247-3p

**Fig 5:**
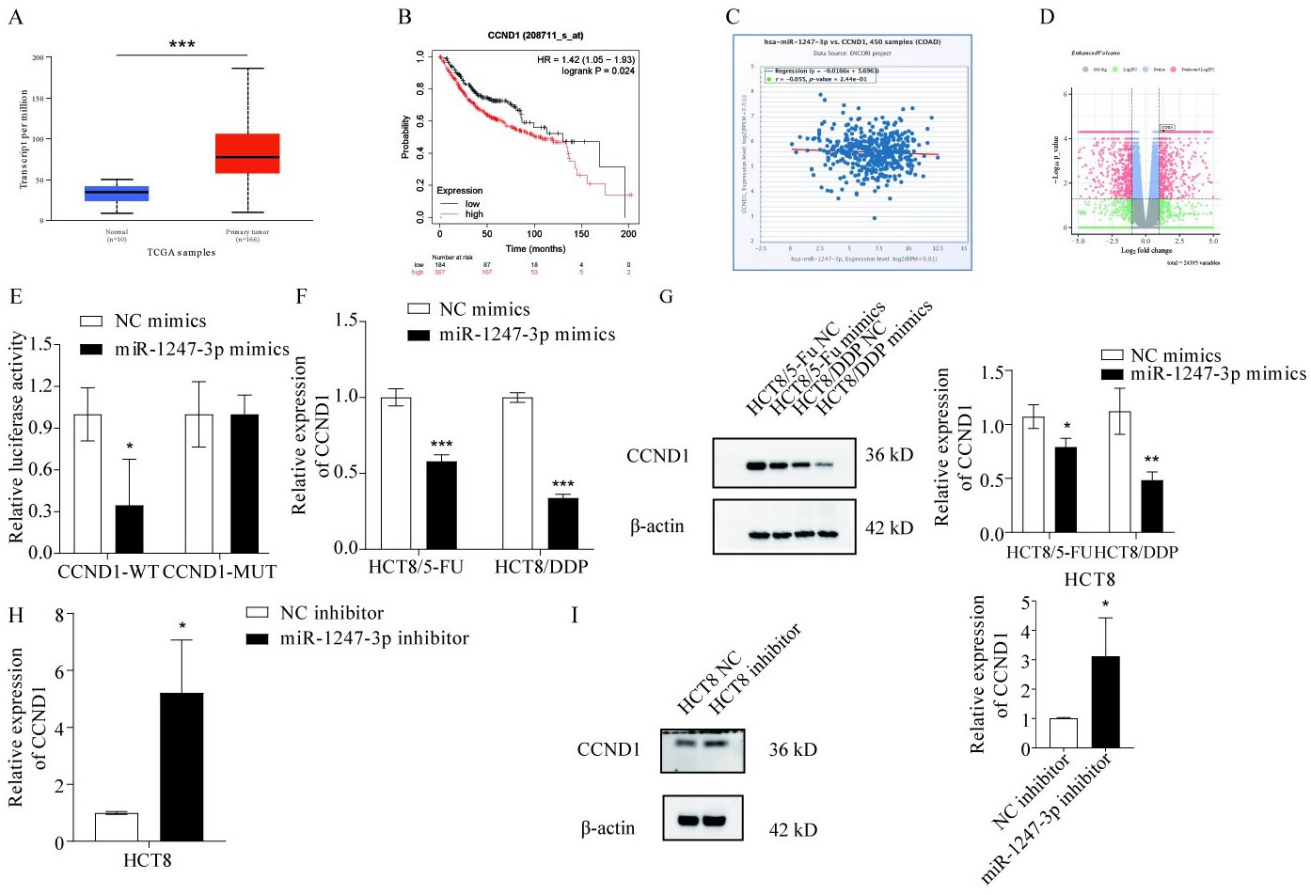
CCND1 is a target gene of miR-1247-3p. (A) and (B) According to the UALCAN and Kaplan-Meier Plotter databases, CCND1 is expressed at high levels in CRC tissues and is associated with prognosis. (C) According to the ENCORI database, there is a certain negative association between CCND1 and miR-1247-3p. (D) Based on the NCBI database, CCND1 is upregulated in tissues of patients with CRC with drug resistance. (E) Dual luciferase detection of miR-1247-3p binding to CCND1. (F) and (G) RT-qPCR and western blotting were used to detect the changes in CCND1 expression in HCT8/5-Fu and HCT8/DDP cells after transfection with miR-1247-3p mimics. (H) and (I) RT-qPCR and western blotting were used to detect the changes in the CCND1 expression in HCT8 cells after transfection with miR-1247-3p inhibitor. *P<0.05, **P<0.01, ***P<0.001. miR, microRNA; CRC, colorectal cancer; 5-Fu, 5-fluorouracil; DDP, cisplatin; CCND1, cyclin D1; RT-qPCR.

The mirDIP (https://ngdc.cncb.ac.cn/databasecommons/database/id/8480) and mirpathdb (https://mpd.bioinf.uni-sb.de/) bioinformatics databases were used to predict the target gene of miR-1247-3p. According to literature review through Pubmed library, STAT5A, XIAP, HMGB1, CXCL12, CCND1, MACC1, SPHK2 were screened as possible targets of miR-1247-3p. However, sequencing results showed that only CCND1 was highly expressed in HCT8/5-Fu (S2A Fig) and HCT8/DDP (S2B Fig) compared to the parental strain. Additionally, the expression of CCND1 in CRC was assessed using the UALCAN database (Fig 5A, P<0.001). The impact of CCND1 on the prognosis of patients with CRC was also evaluated through an analysis available on a specific website (Fig 5B; Kaplan-Meier plotter). The correlation between CCND1 and miR-1247-3p in CRC was explored using another online resource (Fig 5C; starBase or ENCORI: Decoding the Encyclopedia of RNA Interactomes). An analysis of the NCBI dataset revealed differences in CCND1 expression between patients with either drug-resistant or -sensitive CRC (Fig 5D). These analyses collectively indicated that CCND1 showed increased expression in patients with drug-resistant CRC and was inversely correlated with miR-1247-3p. To further substantiate these findings, WT and MUT plasmids were constructed for the 3’ UTR of CCND1 mRNA, the CCND1 plasmid was transfected with the miR-1247-3p plasmid into 293FT cells, and it was verified that miR-1247-3p binds to the WT plasmid, but not to the MUT type through a dual luciferase assay (Fig 5E). Furthermore, HCT8, HCT8/5-Fu and HCT8/DDP cells were transfected with miR-1247-3p inhibitor or mimics, and changes in CCND1 expression were assessed via RT-qPCR (Fig 5F and 5H) and western blotting (Fig 5G and 5I). The mRNA and protein expression results aligned with predictions, reinforcing that miR-1247-3p could decrease chemoresistance in CRC cells by targeting and inhibiting CCND1.

### Downregulation of CCND1 inhibited drug resistance in CRC cells

**Fig 6:**
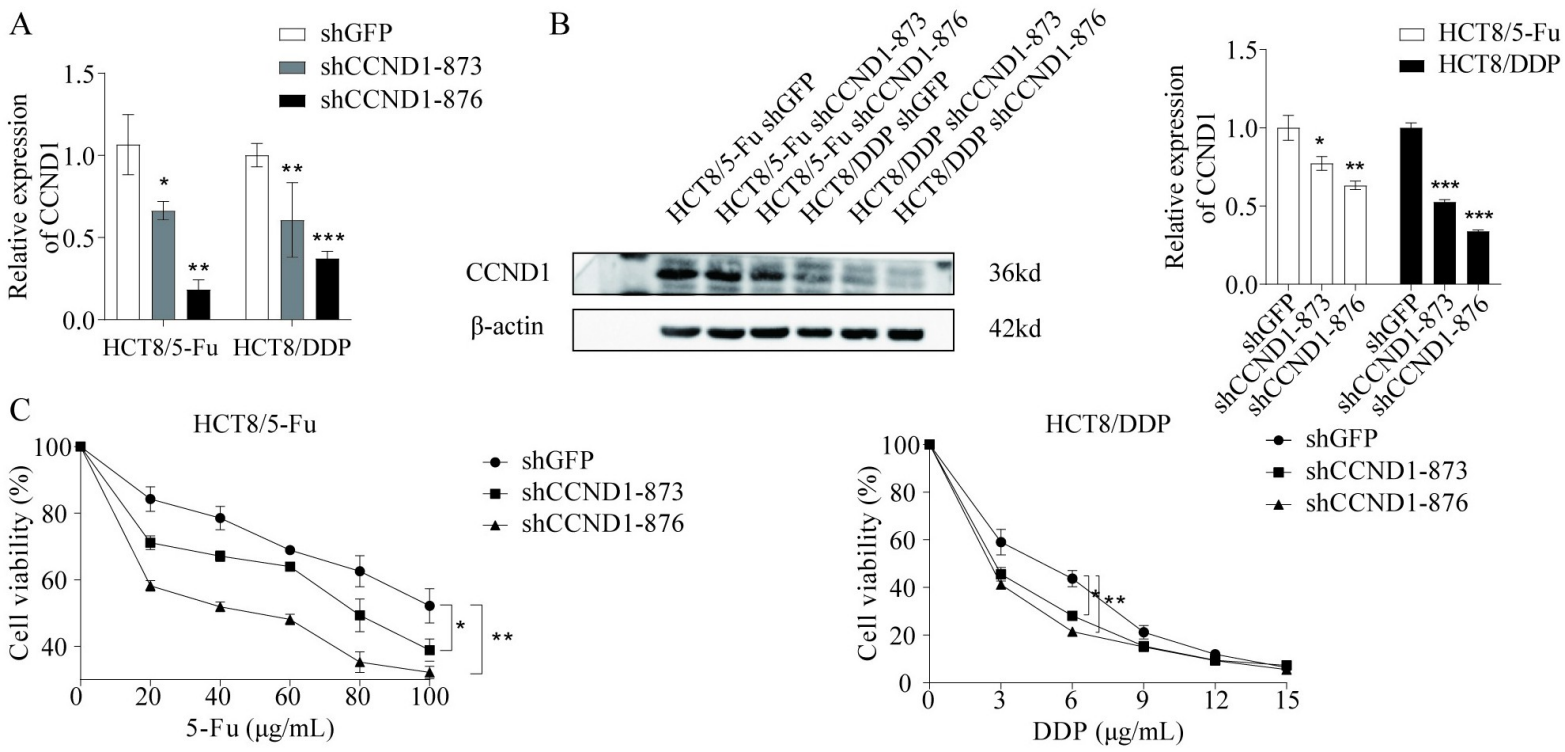
CCND1 promoted chemoresistance in CRC. (A) and (B) RT-qPCR and western blotting were used to investigate expression after knockdown of CCND1. (C) Changes in cellular chemosensitivity after knockdown of CCND1. *P<0.05, **P<0.01, ***P<0.001. CRC, colorectal cancer; CCND1, cyclin D1.

To further validate that CCND1 promotes the resistance of CRC cells to chemotherapeutic drugs, the shCCND1 plasmid was constructed, and the expression of CCND1 was inhibited by lentiviral infection of HCT8/5-Fu and HCT8/DDP, and the knockdown efficiency was detected by RT-qPCR and western blotting (Fig 6A and 6B). The results showed that the sensitivity of CRC-resistant HCT8/5-Fu and HCT8/DDP cells to chemotherapeutic drugs was reduced after the reduction of CCND1 expression (Fig 6C).

## Discussion

miR-1247-3p has a dual role in cancer; it does not only act as a cancer promoter, but also as a cancer suppressor with complex functions in different types of cancers. It has been reported that miR-1247-3p inhibits CRC metastasis through modulated integrin/FAK axis, and it targets STAT5A to inhibit migration and drug resistance in LUAD [22, 23]. However, in oral squamous cell carcinoma and BC, miR-1247-3p promoted cancer development and metastasis [24, 25].

CCND1, a key mediator of cell cycle progression, is a major protein involved in cell cycle regulation and plays a crucial role in the pathogenesis of cancer [26]. Amplification and/or overexpression of CCND1 is frequently found in a variety of cancers [27]. The presence of a large number of mutations in CCND1 is closely related to cancer onset, progression, prognosis and treatment [28, 29]. Numerous studies have shown that dysregulation of CCND1 isoform expression affects various features of cancer [30]. Currently, a large number of studies have shown that overexpression of CCND1 leads to chemoresistance in various malignant tumors, such as testicular, BC, prostate and ovarian cancer [31]. Studies have shown that CCND1 is an important target for several miRNAs regulating cancer development and drug resistance. In colorectal cancer, miRNAs are able to influence the sensitivity of colorectal cancer cells to chemotherapeutic drugs by regulating CCND1 [32, 33], in non-small cell lung cancer, miR-495-3p and miR-545-3p inhibit cisplatin resistance in non-small cell lung cancer by suppressing CCND1 expression [34, 35], and in hepatocellular carcinoma, CCND1 suppresses 5-Fu sensitivity in hepatocellular carcinoma by promoting hepatocellular carcinoma stem cell differentiation to inhibit 5-Fu sensitivity in hepatocellular carcinoma [36]. All of the above studies indicate that the aberrant expression of CCND1 is regulated by miRNAs, but how CCND1 affects chemoresistance in tumor cells has not been elucidated, so exploring the downstream mechanisms by which CCND1 affects chemoresistance in tumor cells is crucial for reversing chemoresistance in tumor cells.

In the present study, the role of miR-1247-3p in CRC drug resistance was explored by transfecting miR-1247-3p mimics and NC mimics into drug-resistant CRC cell lines. The results revealed a significant decrease in miR-1247-3p expression in drug-resistant CRC tissues and cells. Following transfection with miR-1247-3p mimics, there was a notable increase in miR-1247-3p expression and a corresponding decrease in drug resistance among the drug-resistant cells. Furthermore, the overexpression of miR-1247-3p was associated with a significant increase in apoptosis within the drug-resistant cell population and the induction of cell cycle arrest in the G2/M phase, collectively leading to the inhibition of cell proliferation. These findings highlight the potential of miR-1247-3p as a therapeutic target for overcoming drug resistance in CRC.

These findings indeed highlight the crucial regulatory role of miR-1247-3p in CRC chemoresistance. The reduced expression of miR-1247-3p in drug-resistant cells suggests that its downregulation may contribute to the development of drug-resistant cell populations. However, this resistance can be effectively reversed through the overexpression of miR-1247-3p, leading to a significant reduction in cell proliferation, increased rates of apoptosis and enhanced sensitivity to chemotherapeutic agents. Further investigations have also revealed that the upregulation of miR-1247-3p inhibits CCND1 expression in drug-resistant cells.

Therefore, the interaction between miR-1247-3p and CCND1 could be a potential therapeutic target for overcoming drug resistance in CRC. The current study provides valuable insights into the molecular mechanisms of drug resistance and opens up new avenues for the development of effective therapeutic strategies [33, 37]. By suppressing the expression of CCND1, miR-1247-3p may disrupt the normal progression of the cell cycle, leading to cell cycle arrest in the G_2_/M phase. This could be a key mechanism governing chemoresistance in CRC. The results of the present study have significant clinical implications. Firstly, miR-1247-3p emerges as a promising therapeutic target capable of effectively mitigating resistance in CRC cells to commonly used chemotherapeutic agents through its upregulation. Secondly, targeted inhibition of CCND1 could also represent a viable strategy for tackling drug resistance in CRC. Further exploration into the precise role of CCND1 in drug resistance mechanisms and its interactions with miR-1247-3p will provide further insights into the mechanisms underlying drug resistance in CRC. This could pave the way for the development of more effective therapeutic strategies for CRC treatment.

While the current study provides valuable insights into the role of miR-1247-3p in drug resistance in CRC cells, there are several limitations that need to be acknowledged. The precise regulatory mechanisms of miR-1247-3p remain unknown. Future research should explore the potential interactions between miR-1247-3p and other relevant signaling pathways to fully elucidate its role in drug resistance. The present study primarily relied on *in vitro* experiments. The findings have not yet been validated in animal models or clinical samples. Therefore, future studies should aim to confirm these findings in more complex biological systems and clinical settings. Despite the aforementioned limitations, the current study highlights the potential therapeutic value of miR-1247-3p and CCND1 in CRC treatment, paving the way for future research in this area.

The present study revealed the role of miR-1247-3p in reducing the resistance of CRC cells to 5-Fu and DDP by inhibiting CCND1 expression. This finding introduces a new perspective into the investigation of drug resistance in CRC and lays a theoretical foundation for exploring innovative therapeutic approaches that leverage miR-1247-3p and CCND1 as potential targets. Continuing research efforts will further reveal the complex mechanisms underlying drug resistance in CRC, thereby providing enhanced insights for clinical treatment strategies. The current study contributes to our understanding of CRC, and offers promising avenues for future research and treatment development.

## Supporting information

S1 Fig Effect of miR-1247-3p on survival in colorectal cancer patients. Impact of miR-1247-3p in the TCGA database on survival of COAD patients.
(TIF)

S2 Fig RNA-sequencing data screening for miR-1247-3p target genes. Using the parental strain HCT8 as a control, the expression changes of STAT5A, MACC1, SPHK2, XIAP, and HMGB1 were not significant in HCT8/5-Fu (S2A Fig) and HCT8/DDP (S2B Fig). CCND1 was significantly highly expressed in HCT8/5-Fu and HCT8/DDP.
(TIF)

S1 Table Introduction to the dataset. (TIF)

S2 Table RT-qPCR Primer. (TIF)

S3 Table miR-1247-3p plasmid. (TIF)

S4 Table shCCND1 plasmid. (TIF)

S5 Table Dual luciferase primer. (TIF)

S1 Raw images (TIF)
