# Supplementary figures and images for: miR-1247-3p regulation of CCND1 affects chemoresistance in colorectal cancer
Source: PLoS One. 2024 Dec 31;19(12):e0309979. doi: 10.1371/journal.pone.0309979 (PMC11687890; doi:10.1371/journal.pone.0309979)

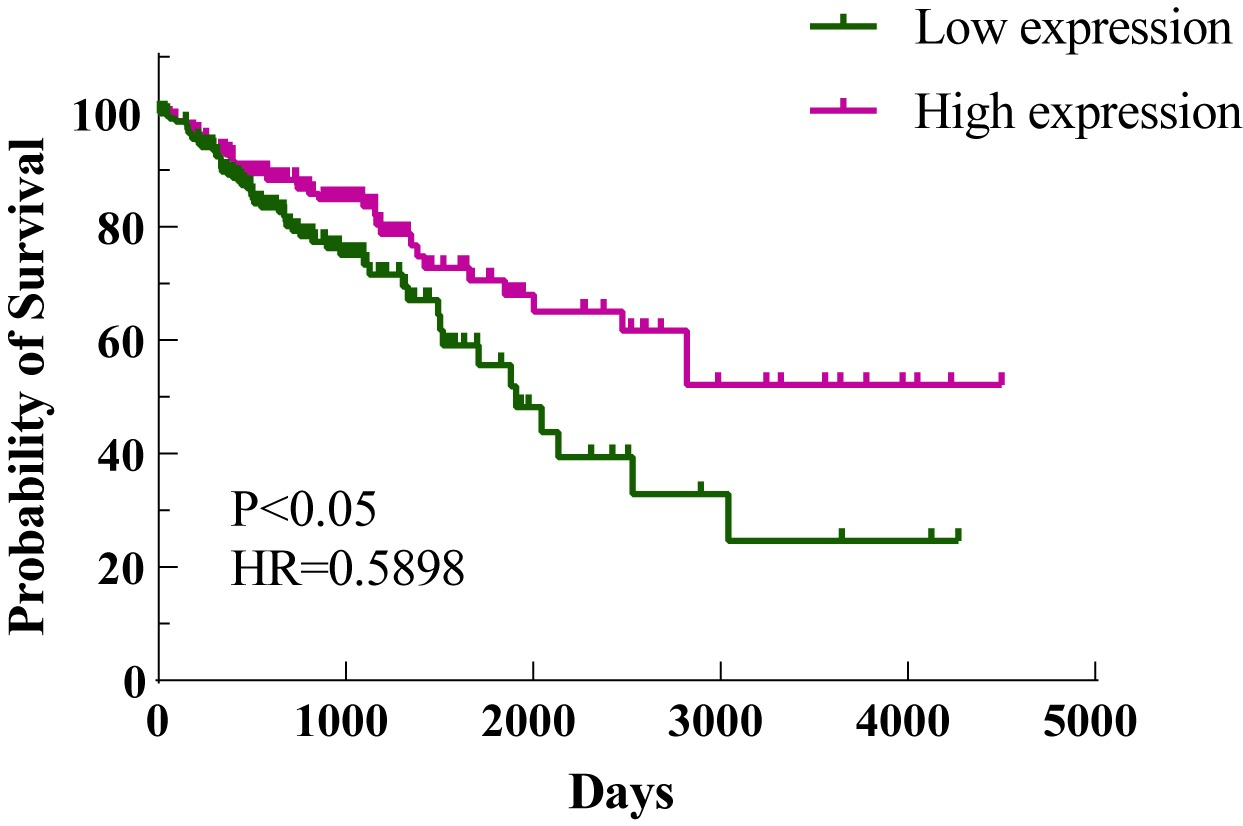

Supplement: S1 Fig — Impact of miR-1247-3p in the TCGA database on survival of COAD patients. (TIF) [file pone.0309979.s001.tif]

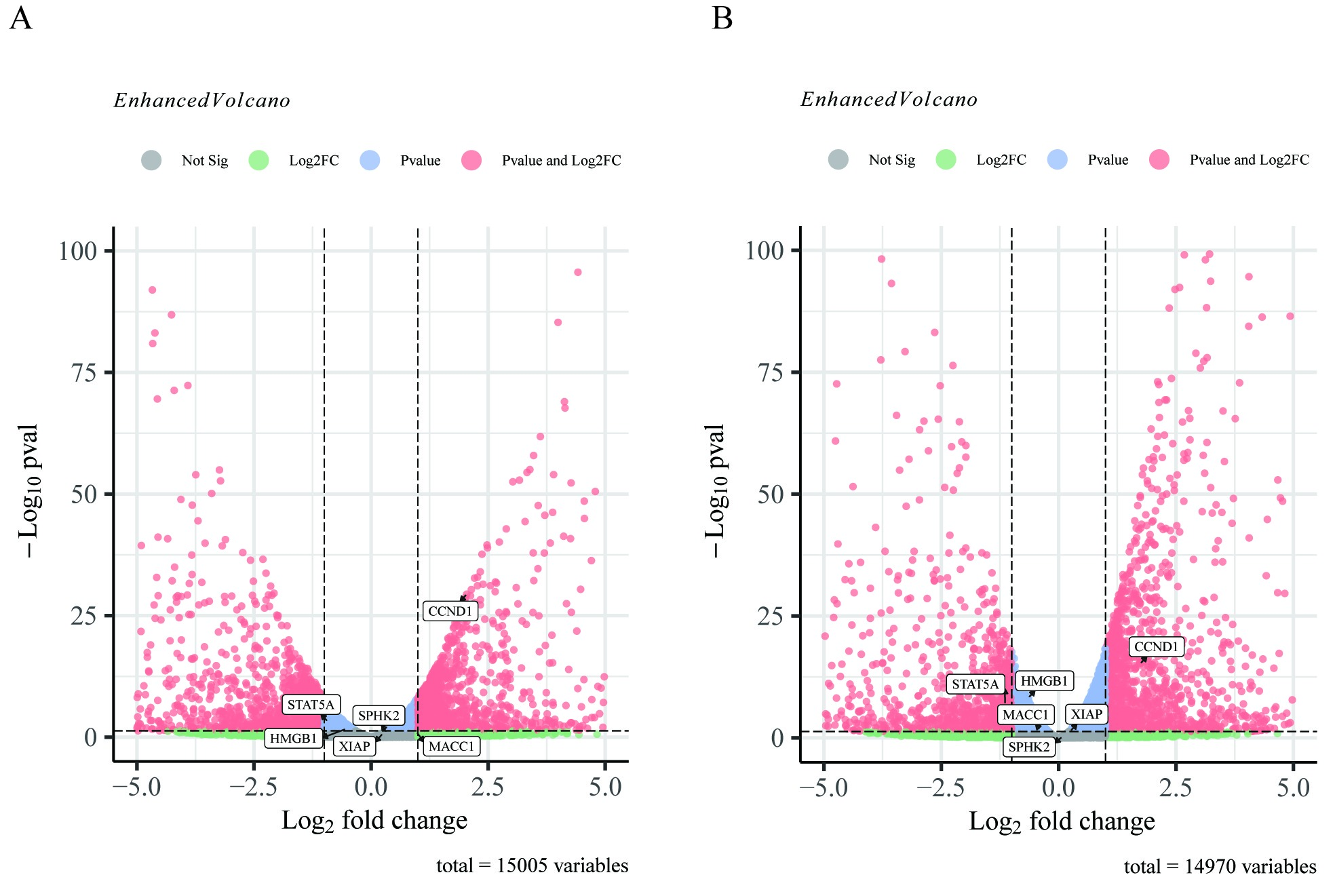

Supplement: S2 Fig — Using the parental strain HCT8 as a control, the expression changes of STAT5A, MACC1, SPHK2, XIAP, and HMGB1 were not significant in HCT8/5-Fu (S2A Fig) and HCT8/DDP (S2B Fig). CCND1 was significantly highly expressed in HCT8/5-Fu and HCT8/DDP. (TIF) [file pone.0309979.s002.tif]

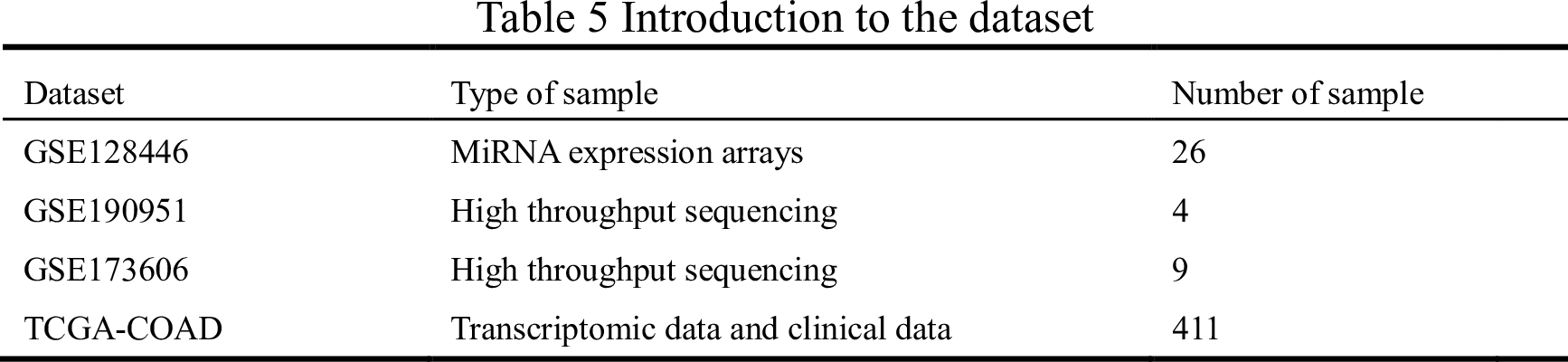

Supplement: S1 Table — (TIF) [file pone.0309979.s003.tif]

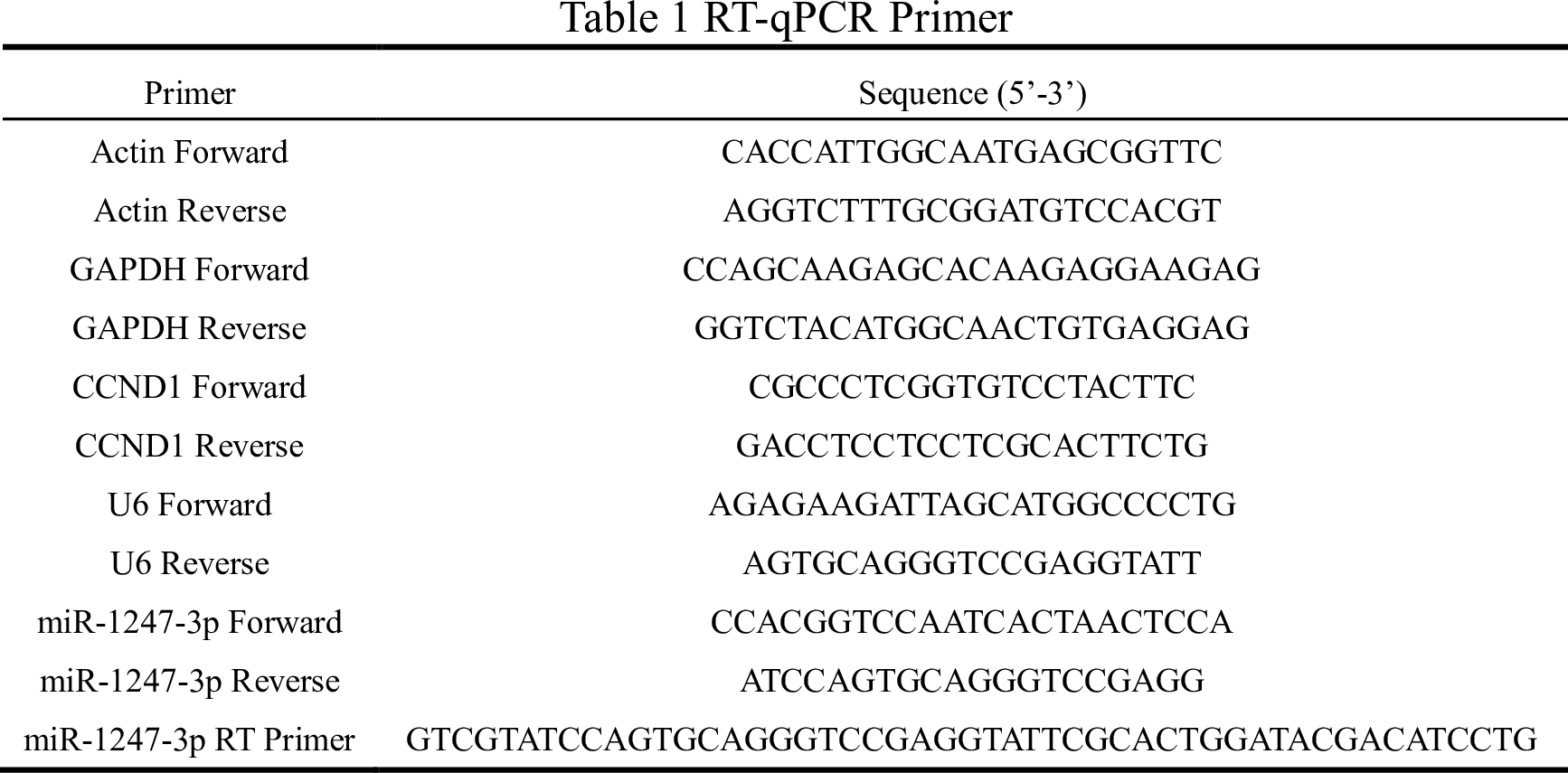

Supplement: S2 Table — (TIF) [file pone.0309979.s004.tif]

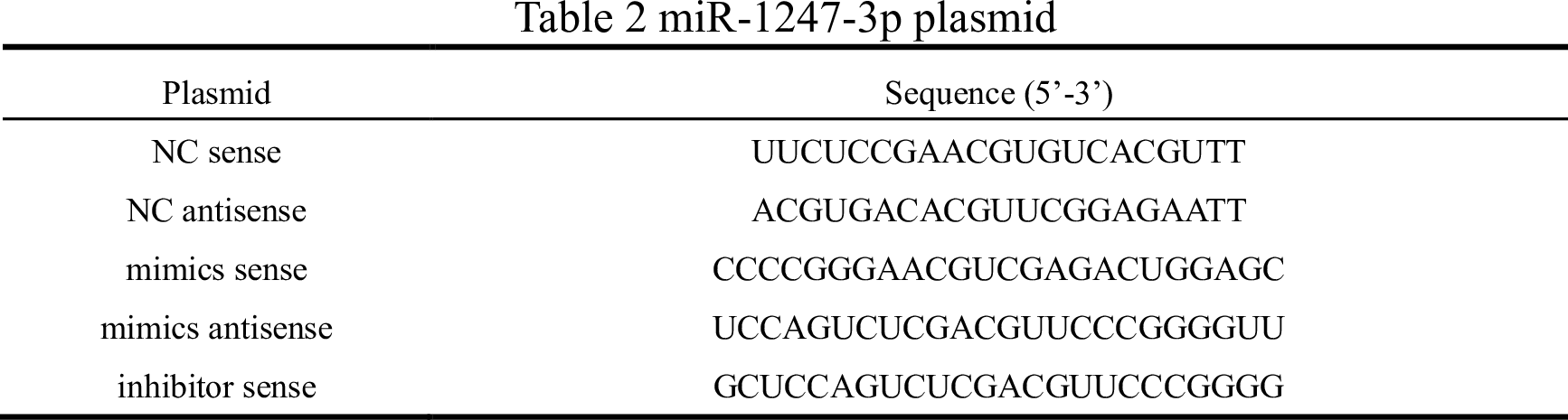

Supplement: S3 Table — (TIF) [file pone.0309979.s005.tif]

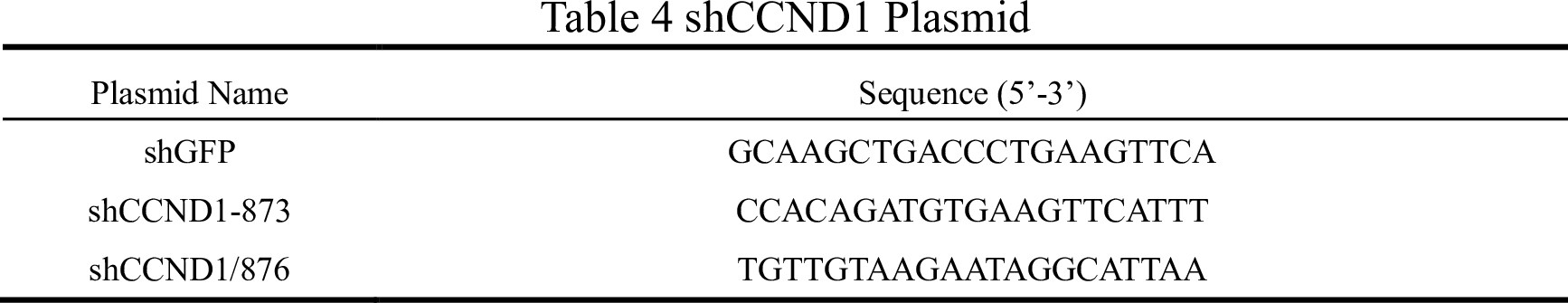

Supplement: S4 Table — (TIF) [file pone.0309979.s006.tif]

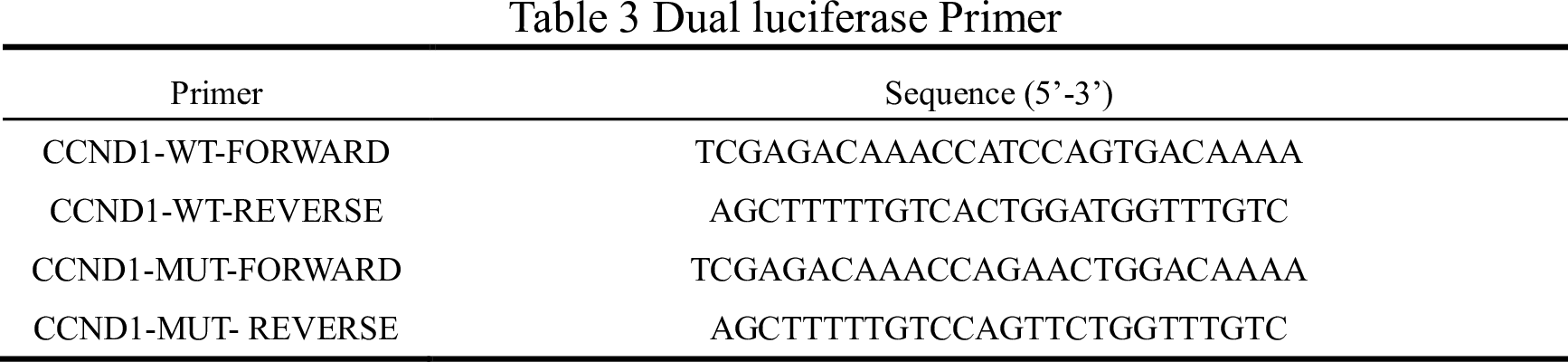

Supplement: S5 Table — (TIF) [file pone.0309979.s007.tif]

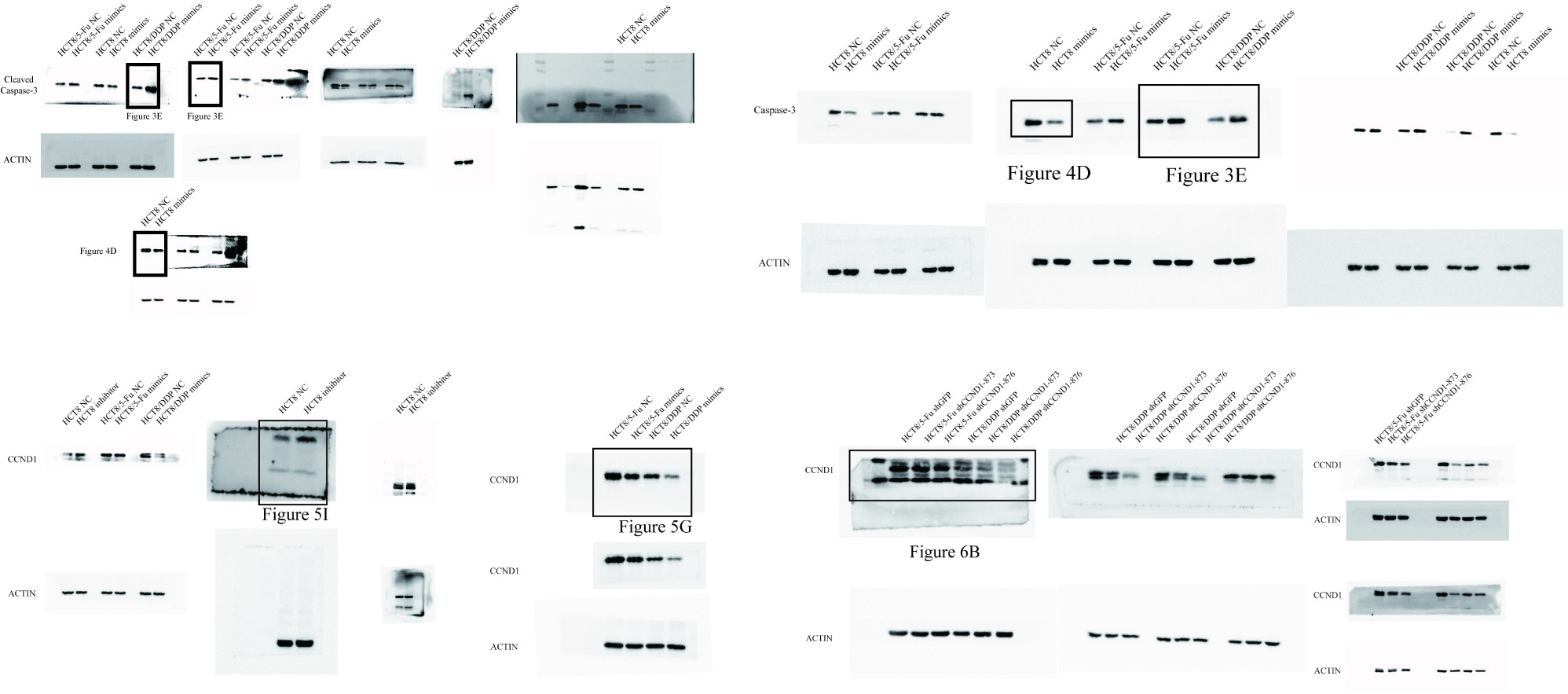

Supplement: S1 Raw images — (TIF) [file pone.0309979.s008.tif]
